# Messaging Preferences about the COVID-19 Vaccine among Adults in Eastern North Carolina

**DOI:** 10.1007/s10900-024-01396-9

**Published:** 2024-09-05

**Authors:** Abby J. Schwartz, Alice R. Richman, Essie Torres

**Affiliations:** 1 School of Social Work, East Carolina University, Greenville, NC USA; 2 Department of Health Education and Promotion, East Carolina University, Greenville, NC USA; 3 Office of the Vice Chancellor for Research, University of North Carolina, Chapel Hill, NC USA

**Keywords:** COVID-19, Health disparities, Vaccine hesitancy, Vaccine messaging, Rural

## Abstract

Racially and ethnically diverse populations and individuals residing in rural areas were disproportionally impacted by the coronavirus pandemic, and Eastern North Carolina (ENC) is one region where such impacts were apparent. To understand at-risk individuals’ perceptions and hesitancy to COVID-19 vaccines and the preferred means of receiving vaccination-related messages, we conducted four qualitative focus groups (*N* = 40) with diverse rural ENC residents. The analysis of the focus group transcripts revealed five themes: (1) reasons people trusted the COVID-19 vaccines, (2) reasons people mistrusted the COVID-19 vaccines, (3) the best means to deliver messages regarding COVID-19 vaccination, (4) the individuals trusted most to deliver such messages, and (5) the decisions people made regarding whether to get vaccinated and how that was connected to God. By incorporating participant perspectives and preferences in receiving messaging into campaigns, there is a potential for greater vaccine uptake.

## Introduction

The coronavirus pandemic caused morbidity and mortality on a large scale, revealing disparities in infection, hospitalization, and death among affected Black, Indigenous, and People of Color (BIPOC) in the US [1], in addition to greater impacts experienced by people living in rural communities. A national cross-sectional survey found that adults in rural communities (remote or metro adjacent) experienced greater anxiety, depression, and monetary difficulties (e.g., timely payment of bills) due to the pandemic as compared to their urban counterparts [2]. Rural US communities also had challenges with meeting the needs of those hospitalized for COVID-19, including limited numbers of intensive care unit beds [3, 4].

North Carolina (NC) is one state with populations that were disproportionally affected by COVID-19. Seventy-eight percent of the counties in NC are characterized as rural (≤ 250 per square mile) and the current study setting a 29-county subregion in eastern NC (ENC) is identified as 93% rural [5]. Approximately 76% of the counties in ENC are designated as Tier 1 (most distressed) [6], and the ENC region is considered a medically underserved area (MUA) with a shortage of health professionals. Twenty-eight of the counties are designated as MUAs in terms of availability of primary care physicians (data not available for one county) [7]. As of 2022, across all 29 counties the average rate per 10,000 population of primary care physicians was 4.83 (median = 0.73) compared to the state rate of 7.42 [8]. Lastly, a greater number of BIPOC residents live in ENC compared to NC (~ 42% versus ~ 38%, respectively) [9].

COVID-19 vaccination rates in ENC remain lower compared to NC overall. The now archived NC Department of Health and Human Services COVID-19 Vaccinations Dashboard noted in April 2023 that 68% of people in the state had at least one dose of the COVID-19 vaccine, and 63% completed the initial series. However, these percentages decrease in ENC counties. For example, in Washington County, 57% of individuals received at least one dose, with 52% completing the initial series [10]. Lower vaccination percentages suggest decreased access to health care and/or COVID-19 vaccine hesitancy.

Vaccination hesitancy has been examined including factors associated with it. In a systematic review, three factors associated with hesitancy included being Black or African American, being above 45 years of age, and having a lower socioeconomic status [11]. Research examining rural settings and COVID-19 vaccine uptake cited reasons for vaccine hesitation including risk and safety concerns such as the novelty of the vaccine, its swift development, side effects both immediate and long-term, and beliefs concerning the effectiveness of natural infection [12, 13].

As a medically underserved, predominantly poor, racially and ethnically diverse, and rural region, it is critical that practitioners and health professionals understand the perceptions ENC residents have about vaccines to inform and educate communities in culturally acceptable ways and promote vaccination uptake. The present research was part of a larger study examining diverse medically underserved communities living in ENC and the reasons for COVID-19 vaccine hesitancy and any structural barriers experienced by this population. Testing and developing COVID-19 messages promoting vaccination were also included in the larger study, with the goal of developing messages by community members as opposed to those provided by larger entities (e.g., government). Given the rich focus group data collected, this paper provides focus group participant perspectives on reasons to trust and mistrust COVID-19 vaccines, and preferred ways to receive messages promoting vaccine uptake.

## Methods

### Participant Selection

Purposive and snowball sampling were used as recruitment methods in the two ENC counties where the focus groups were to be conducted. Given the first and second author’s research and engagement in the ENC community previously, community partners were invited to take part in the research and asked to invite others who may be interested in participating. Recruitment included disseminating a research flyer by email to community partners and organizations that have contact with community members. The flyer stressed that all thoughts and opinions on COVID-19 vaccines and messages were welcomed. Eligibility included being English-speaking, at least 18 years of age, and residing in one of the 29 counties in ENC. This study received approval from the East Carolina University and Medical Center Institutional Review Board (UMCIRB).

### Data Collection

Four qualitative focus groups (*N* = 40) were conducted with individuals living in rural ENC between August and September of 2022. Discussions were moderated by the first two authors and lasted 1–1.5 h. The focus groups were held at accessible centrally located places for residents. This included a Fire Department (*n* = 10), Senior Center (*n* = 7), private room in a local coffee shop (*n* = 15), and at a soup kitchen for unhoused people and/or those requiring nutritional assistance (*n* = 8).

Following informed consent, participants completed a demographic survey, and then the discussion began. The goal of the focus groups was to collect information regarding COVID-19 vaccine hesitancy and trust, and the development and delivery of acceptable messages regarding COVID-19 vaccination among the focus group attendees. Results from the following areas of the topical guide (Table 1) are presented: (1) discussion of COVID-19 vaccines and reasons for trust and mistrust (Table 1, Topic A), and (2) questions about the best methods of vaccine message delivery and those considered as trusted individuals to deliver such messages (Table 1, Topic B). Participants were provided with a $50 Visa gift card at the end of the focus group discussion to thank them for their participation.

**Table 1:** Focus group topical guide

A. Discussion of COVID-19 Vaccines
1. What do you think of when I say the words ‘COVID-19 vaccine’? (word association) 2. What are some things you’ve heard about the COVID-19 vaccines that make people trust them? 3. What are some things you’ve heard about the ‘COVID-19 vaccines’ that make people *not* trust them?
B. **Message Delivery**
1. What method of message delivery would be best for you and why? o *Probe*: newspaper, radio, social media, doctor, text message, emails etc. 2. What method of message delivery would work best for those in your community? 3. Who would people trust the most to deliver the messages? o *Probe*: doctor, celebrity, church leader, parent, family member, friend.

### Data Analysis

The transcribed recordings were analyzed using Braun and Clarke’s (2006) approach to thematic analysis [14] and inductive and deductive coding was also used [15]. For deductive coding, codes were developed prior to the analysis and based on the interview guide questions. For example, such codes included reasons to trust COVID-19 vaccines, reasons not to trust COVID-19 vaccines, best method message delivery, and people trusted to deliver messages. Inductive codes emerged from the data and were not developed based on the interview guide but rather on the patterns of statements made by participants in the transcripts.

The lead author (AS) led the thematic analysis process and began by reading all transcripts. Following the initial read, AS went back and began using the predefined codes developed based on the interview guide and open coded words and phrases of interest that emerged from the data in each transcript. AS then entered the coding of each transcript into NVivo (Version 12) [16]. Once this was completed, AS printed the transcript data associated with each code for review. In this second review, AS collapsed overlapping codes into what became initial themes. AS then recoded the data in NVivo given the themes developed. Then AS printed out each theme for the second author (AR) to review and determine alignment and agreement with the list. Any disagreements were resolved with discussion and consensus was met.

Trustworthiness was established in various ways through the data collection and analysis process [17, 18]. An audit trail was maintained throughout the data collection and analysis process including coding accuracy, analysis process notes, and field notes completed by the authors following each focus group. The authors also were engaged in the region for a prolonged amount of time. Prior to the present study, given their research foci on health disparities and other work done within the region, the authors had already established rapport with some stakeholders and community members, and this further strengthened the data collection process.

## Results

Table 2 provides the demographic characteristics of the focus group participants. Participants were an average of 57.1 years of age and were predominantly Black or African American (80%). Approximately 36% of the participants reported household incomes at or below $19,999, with the next largest percentage (25.6%) including those with household incomes of $70,000 or higher. 35% of the participants were employed full time, and 45% were retired or on disability. 45% of the participants had a college or graduate school degree, 27.5% had a high school diploma/GED or some high school education, and 27.5% had some college or trade school or associate degree.

**Table 2:** Demographic characteristics of participants (*N* = 40)

Characteristic	Mean (SD), Range	%
Age (*n* = 36)		
Female	57.1 (11.9), 35–81	77.5
Income (*n* = 39)		
<$10K – 19,999 K 20 K- 29,999 K 30 K-39,9999 K 40 K-49,999 K 50 K-59,999 K 60 K-69,999 K 70 K+ Don’t know Prefer not to answer		35.9 5.1 5.1 7.7 2.6 7.7 25.6 2.6 7.7
Race		
White Black or African American Other^a^ Prefer not to answer Ethnicity: Hispanic/Latino (*n* = 36)		10.0 80.0 7.5 2.5 2.8
Educational Attainment		
Less than HS/HS/GED Some college or trade school Associate degree College degree Graduate School		27.5 17.5 10.0 22.5 22.5
Employment Status		
Full time (FT) Not working Retired On disability Other^b^ Prefer not to answer		35.0 5.0 20.0 25.0 12.5 2.5

Note. ^a^White & Black (*n* = 2) and Black Indian (*n* = 1); ^b^Retired & FT (*n* = 1),

FT & Part-time (*n* = 2), Multiple Part-time & Retired (*n* = 1), Retired & On Disability (*n* = 1)

### Focus Group Themes

The following themes emerged during the analysis process of the focus group transcripts. Note that in the succeeding sections FG1, FG2, FG3 and FG4 are used to denote participant statements from the first, second, third or fourth focus group, respectively. Five themes emerged from the analysis and included the following: (1) Reasons to trust the COVID Vaccines, (2) Reasons not to trust the COVID vaccines, (3) Best methods of delivering messages about COVID vaccines/getting vaccinated, (4) Most trusted individuals to deliver messages, and (5) Decisions regarding vaccination and the connection to God. Table 3 provides a listing of each of these themes and associated subthemes if applicable.

**Table 3:** Themes and subthemes

Themes & Subthemes
1. Reasons to Trust the COVID vaccines • Will not get as sick if get COVID • To protect others • Interactions with trusted individuals
2. Reasons not to trust the COVID vaccines • Vaccine came out too soon • Vaccine side effects • Beliefs about the vaccine: why it exists and what it can do to you
3. Best Methods of Message Delivery
4. Most Trusted People to Deliver Messages • Word of mouth • Personal testimony and inclusion of “celebrities”
5. Decisions regarding vaccination and the connection to God

### Reasons to Trust the COVID Vaccines

The first theme pertains to why respondents or others in their community trust the COVID-19 vaccines. Within this theme, three subthemes emerged including *will not get as sick if contract COVID and have been vaccinated*,* get vaccinated to protect others*,* and interactions with trusted individuals.*

#### Will not Get as Sick if Get COVID

Individuals expressed the importance of getting the vaccine to avoid getting COVID, or, if to prevent themselves from being sicklier if they contracted the virus. One person described how the vaccine helped those who did get COVID, “*… [people] that were having complications like through their systems…it [the vaccine] helped them to not get as sick… the ones who were able to fight it*,* it gave ‘em the bacteria they needed…” (FG4).* The perspective of what could happen if someone did not elect to get vaccinated was also discussed. *“The people that didn’t take the vaccine were just like really*,* really sick…If you take the vaccine*,* it wouldn’t keep you from getting COVID*,* but you wouldn’t get as sick. That’s why I took…”* (FG1). This was reiterated by a participant in focus group two who stated, *“people who probably had the vaccine but then contracted or contacted COVID but overcame the*,* like the*,* the disease and little to tell the story…or it wasn’t as bad as the person who did not get the Vaccine*.*”* A focus group three participant felt it critical to get vaccinated to prevent them from dying, *“I have*,* you know*,* conditions. So*,* I feel like it would be safe. So*,* if I catch it and I did catch it and it was not as deadly like it could be…”*.

#### To Protect Others

Protecting others, friends, or family, or community members was a key reason why people chose to trust the vaccines. As described by one parent, *“I wanted to get my vaccine…My son is immunocompromised*,* and I knew that if I got it [COVID-19]*,* that he couldn’t get a vaccine yet”* (FG2). Those who did not initially get vaccinated changed their mind after an experience they had with a family member. One person said she was not going to get vaccinated, but her grandchildren convinced her to do so.

*At first*,* I was a little skeptical and even my grandsons came and said*,* “Grandma*,* will you stay out in the street and go get your vaccine?” And I’m like*,* “Let me stay out in the street and go get my vaccine” (FG2).*

Another participant recalled how painful it was to miss Christmas with her son due to his COVID-19 infection prior to vaccinations being available. *“I saw my son had COVID…and we couldn’t celebrate Christmas together. I could only see him through the door. And I saw the tears in his eyes ‘cause we couldn’t touch each other”* (FG3).

In addition to protecting family members as described above, individuals also expressed that getting vaccinated was part of their civic duty. A participant explained their understanding of how COVID could be spread and the reason why it was important they got vaccinated *“…you can carry and pass [COVID] along. But I don’t want anything to be passed along. I felt like my body is healthy enough and my faith is strong enough that whatever I inject myself with*,* I could fight”* (FG3). In other cases, fulfilling this civic duty was not a choice and people were mandated to get vaccinated to protect themselves and others. In the fourth focus group a participant explained that “*for my job it was required…you would lose your job.”*

#### Interactions with Trusted Individuals

Interactions people had with those they trusted was instrumental in their getting the COVID-19 vaccine. For some participants, the trusted individual was a doctor, however, the doctor was someone who had cared for them for a long time who had they longstanding trust and rapport with. *“That’s one doctor in my life that I really trust*,* and I know he wouldn’t lead me the wrong way…but if it would’ve been another doctor…I would’ve said ‘nuh uh’”* (FG1). This was reiterated by another participant, *“I know he [doctor] wouldn’t lead me the wrong way…I trust him*,* he cared for me and not for their Medicaid money. And I’ve been with him ever since ‘92. He brought me back to life”* (FG1).

A statement by a participant who was nurse in charge of giving people the vaccine further explained the importance of trust and long-term relationships.

*… the people I grew up with were most excited about getting the vaccine because I was giving it to them. Like one of their classmates who now is a nurse who they can trust… somebody they can truly relate with…I gave it [to] probably all of my high school classmates…they all heard you know they were giving shots and that you know*,* [participant name] was giving it. (FG2)*

### Reasons Not to Trust the COVID Vaccines

There were several subthemes that described why participants did not trust COVID-19 vaccines. These subthemes included *the vaccine came out too soon*,* side effects from the vaccines*,* and beliefs about what the vaccine could do to your body*.

#### Vaccine Came Out too Soon

The perceived swift development and research of the vaccines was mentioned repeatedly. In focus group one, a participant explained how, *“People didn’t trust it [the vaccine] because how quick it came about. How quick it was formulated.”* A participant expanded on this by stating, *“I think that [the vaccine] was ready so quickly…there wasn’t enough research involved.”* (FG4). Unease concerning time and research was reiterated further suggesting it was not read for dissemination, *“Apprehension due to the gestation period of its development. The longevity or lack thereof of it being developed. Or it was ready to be given out.”* (FG3).

Concerns were also voiced about vaccine safety and swiftness combined with which pharmaceutical company developed it. “*Johnson & Johnson got ‘pulled.’ So*,* the people that took Johnson & Johnson previous[ly]… where did that leave them? What kind of headspace to leave them in*,* you know*,* what kind of safety net did they have?”* (FG1). Given the lawsuit the company was engaged in, this decreased trust of the vaccine even further. A participant described this as follows:

*…another concern that I had…was one of the companies that was selected to make the vaccine. Johnson & Johnson*,* the ones that are in lawsuit because of cancer from baby powder…I don’t think that’s right ‘cause I don’t think that company revealed it…the fact that through the research they knew that the type of…powder…causes cancer. So that led me not to trust that”* (FG4).

#### Vaccine Side Effects

Many participants reported hesitating getting vaccinated because of potential side effects. “*People were concerned about the side effects. I wanted to wait to get it because I wanted to make sure”* (FG4). The side effects participants experienced following vaccination was also discussed by the participants, such as, “*I had a horrible time with both of my vaccinations. I had these swollen glands. I had everything*,* all of the side effects”* (FG1). Death was another side effect/possible outcome discussed. A participant in the first focus group explained, *“One of the most detrimental things I saw…was that when medical professionals would have like underlying conditions and then would get the vaccine…then passed away”* and, *“people that got the vaccine*,* and [they] still died….so it doesn’t work…”* (FG4). Seeing/knowing others who experienced severe side effects exacerbated mistrust and fear. One participant described an experience she had when the COVID vaccination was initially released:

*I volunteer at my job to go to the clinics … [when the] outbreak first came*,* and the vaccination started I was in the building*,* working…helping people get signed up for vaccination. They was [sic]getting administered…and I seen [sic] so many in the beginning*,* people falling out*,* had seizures … So*,* for myself personally*,* that …put a damper on things…scared me* (FG1).

Another participant described how the debilitation of a friend’s daughter could only be attributed.

to the vaccine:

*I have a friend; her daughter went to school…she play [sic] softball on the team where she got a scholarship and they were saying that the kids had to be vaccinated… the child took the vaccine and then she can’t walk anymore…it can’t be something she had before. It had to be in that. So*,* the vaccine*,* it can do different things to different people…and that’s the scary part.* (FG3).

#### Beliefs About the Vaccine: Why it Exists and What it can do to you

Another theme related to distrust was beliefs about the ulterior motives concerning the creation of the COVID-19 virus and vaccines and what they could do to your body as opposed to being created to save lives. A participant in the second focus group stated, *“I heard population control…getting rid of the elderly…people who didn’t have like a longer lifespan to get rid of those people …*” Another participant shared her husband’s thoughts and how he said, *“‘the strong survive’…the ones that pass or didn’t take the vaccine*,* he was like*,* ‘you know*,* only population control’”* (FG2). This was expanded upon by a participant in the fourth focus group and tied to what they believed was the larger greater purpose at the governmental level.

*It was a whole plot…a new world order thing. That the government has been…proceeding all these years to depopulation his own American people. Government don’t care. Filthy rich don’t care…. we just*,* uh lower levels of things…if you read into certain things and…do a little research*,* you start realizing that all this was set up for a reason.*

In addition to depopulation, beliefs about how one’s body is modified by the vaccine were discussed at length. One person in focus group three reported what her niece told her,

*…everybody that had shots*,* if you put your cell phone to your arm*,* that sticks to your arm…[people laughing in background] Ya’ll laughing*,* but it’s for real…that magnetism in your blood… Cause my niece had did [sic] it … I said*,* “Lord*,* and I done took the shot.” But for real it did stick to her arm.*

Another participant confirmed that they heard about magnetism and the vaccine:

*I heard the same thing. My son called me one day and was sitting in the back seat and a magnet stuck*,* and I said’ “try it in the other arm” …he said*,* “no*,* don’t work on the other arm. It works on the arm that you got the shot in.”*

A participant in the fourth focus group explained how, *“It’s to change the body system DNA to RH*,* to the RH DNA*,* something like that. But it’s to change the whole-body system…”*.

### Best Methods of Message Delivery

As indicated in Table 1, participants were asked what methods of COVID-19 message delivery (e.g., newspaper, radio, text, emails) would be best or most effective for them and/or their community and why. The preferred means of communication varied based on the intended audience. If the target audience was older adults, participants reported that mail, radio (gospel stations), churches, and senior centers were potential places/ways to reach the older populations best. As a second focus group participant explained, “*I said social media and tv because then you’re hitting another generation…some of our older folks don’t [do]…social media things.”* Social media was also noted as a possible positive resource that could bridge generations. *“So*,* if it’s like a hashtag ‘go get vaxxed*,*’ QR code*,* let them register their older folks to get them vaxxed and get an Uber ride all in the same thing to go pick them up”* (FG2).

The church was mentioned as a great way to deliver COVID-19 messages. Some participants’ churches actively discuss and promote vaccination. “*I know our church*,* they talk about COVID practically every Sunday. ‘Make sure you get your COVID shots’” (FG3).* Church announcements were also mentioned as an appropriate means of message delivery and is aligned with what the participant above reported.

News on television and newspapers in print and/or online were perceived as reliable by some and untrustworthy by others to disseminate information about COVID-19 and vaccines. Those who used the news accessed it in various forms including small town newspapers, “*Here in [town name]*,* you know*,* it’s small…the news…and they got that online as well as you can buy a physical copy*” (FG1); or through TV and various stations, *“I go to the news sources. I have DirecTV and so…it gives me CNN and MSNBC and then Fox”* (FG3). However, others did not believe the news would be a reliable source, as it promotes fear among viewers and information is inconsistent and constantly changing.

*I feel like they spread premature information like research…get all the information*,* the real information and facts you need. Then go to the media with it… they create so much fear….and the story changes as it goes and it makes it worse…Like with the CDC*,* you know*,* their stuff just changed so many times…well now I’m not listening.* (FG4)

In addition to creating fear, the news was seen as more focused on selling advertising and was also biased. “*So many people talk about the media. Often that it’s being fake…you don’t know what to believe anymore. Because the media now so much about selling advertising…rather than just giving the straight unbiased news” (FG3).*

### Most Trusted People to Deliver Messages

#### Word of Mouth

As trust was an underlying theme related to message delivery, word of mouth and listening to friends and family members who got vaccinated was perceived as appropriate. A participant in the fourth focus group said, *“You go about word of mouth…well*,* trusted word of mouth. Cause I don’t just listen to any word of mouth. The personal message from someone you trust.”* One person emphasized further upon on the statement above, “*I think your best messaging is the people who are getting it…let them talk about what their experiences are. Peers. Word of mouth*” (FG2). As alluded to in this quote, there was a sense of testimony – hearing from those who can prove that the vaccine worked and, in some cases, saved their life.

#### Personal Testimony and Inclusion of “Celebrities”

Participants reported that personal testimony by individuals who received the vaccine and overcame COVID-19 were critical in messaging. Such statements included: *“…if I told them… I had COVID*,* I was on ICU*,* here are my picture*,* here’s proof… I mean give my testimony to you that it does work”* (FG2). A participant in focus group one succinctly explained, *“If there’s somebody that experienced it theirself [sic]…then I prefer to hear it from them.”* In the context of testimony, a participant described how, *“a lot of people trust celebrities ‘cause they watch ‘em on TV or stuff like that and they follow them a lot…”* (FG4). In the focus group discussions, “Celebrities,” were defined as people who are well known and respected in the community and/or a nationally known but from the ENC region. Such individuals were identified as effective messengers of testimony who can promote vaccine uptake. An example of a regional celebrity was also provided.

*If you ever been through [town]*,* they put up those billboards with different local celebrities on it instead of healthcare workers… people in [town] don’t trust the hospital. So*,* they didn’t go there with healthcare. They went with… [Name of NBA athlete] is from [town]…they put…the local celebrities (FG2).*

In addition to NBA level celebrity status, more local celebrities were discussed with a focus on those who currently live among community members. *“You got like*,* a lot of people just trust their pastors and stuff too ‘cause they live among them. They see them every day”* (FG3). Using pastors was discussed in more detail by a participant who was actively disseminating vaccine information in the community,

*…that’s why we started focusing on black churches…we saw that pastors were directing their shepherds of their flock. And we knew that if we could start with the head*,* they could help direct some of the conversations…*(FG2).

### Decisions Regarding Vaccination and the Connection to God

Individuals’ belief in God and decisions regarding vaccination were inextricably linked. Regardless of the questions in the interview guide, God would emerge as a topic of discussion in each of the focus groups and how for some people their faith in God would be a deciding factor whether to be vaccinated. A participant in focus group two who was working in the community disseminating vaccines explained:

*When we were trying to get people to get their vaccination*,* “well God got me covered” …I’m sitting here looking like*,* “I’m a Christian and I think you’re an idiot*,*” …and they’re looking at me like*,* “I don’t care what you say*,* my God got me covered*,*” and I’m like*,* “my God got me covered as well. But he also gives me the knowledge to be able to do what I’m doing and you’re not making sense.” So*,* a lot of … almost bargaining with people and they’re like … “I trust God*,*” and you don’t wanna step on that line of their faith* (FG2).

A focus group three participant emphasized how God gave humans knowledge and common sense:

*God has also given man common sense… God gave man wisdom and knowledge to create that seatbelt for your safety and wellbeing*,* the same way he gave man wisdom and knowledge to create the vaccine. Whether or not you choose to embrace it…that’s on us. I’m not gonna bash that person who decides not to get the vaccine cause because they’re believing God to cover them. And I’m not gonna embrace the person who’s decided to get it* (FG3).

As noted in the quote above, despite one’s opinion on whether someone should get vaccinated, a decision not to do so should be respected. It was also reported that individuals who got vaccinated and still got the virus felt that their survival could be attributed to God. “*You know*,* even though I did take one shot…I still caught the COVID regardless and I made it through*,* not because of no shot*,* but because of God”* (FG4). A focus group two participant shared the same sentiments about someone that they know who is a truck driver and chose not to be vaccinated. “*…she’s a truck driver… She went from New York to … North Carolina … And that’s what she And if you say*,* ‘Well*,* are you vaccinated?’ ‘God vaccinated me.’”*.

## Discussion

Focus group participants provided various reasons as to why the COVID-19 vaccines could be trusted. Discussions around not getting as sick if they got the virus, getting vaccinated to protect others from getting sick, and having conversations with trusted others who recommended getting the vaccine were all reasons participants felt getting vaccinated was important. The reasons to get vaccinated overlapped with perceptions of who the most trusted people are to deliver vaccine-related messages from word of mouth and by testimony from local celebrities (e.g., health professionals, pastors) or famous individuals (e.g., black athletes) with ties to the region. Similar findings have been found in other research indicating that trusted individuals in and from the community are critical are engaging people in discussing COVID-19 vaccines [19–22]. While we called this “testimony” other research has identified this as storytelling [23]. Trusted sources of information could include church leaders sharing their vaccination experience [19] churches [24] trusted doctors and individuals in the community [19–23]. Thus, engaging such trusted sources is critical in the promotion of COVID-19 vaccinates among racially and ethnically diverse populations.

Concerns about the safety of the vaccines was the most discussed reasons as to why the vaccines could not be trusted. Like prior research focused on racially diverse individuals, reasons such as how “new” the vaccine was, how quickly it was developed, and any type of side effects known and unknown were reasons for mistrust [12, 21, 25, 26]. The participants also had concerns surrounding the pharmaceutical companies that developed the vaccines and this was mentioned in prior research, particularly the lack of information available concerning the research and development phases of the vaccines [20, 24]. In the present study, participants expressed distrust regarding why and how the vaccine was developed. Beliefs regarding its development for population control was included, and a mistrust of the government was noted. These findings are supported by prior research focused on racial/ethnically diverse populations who noted the same concerns including statements such as being used as “guinea pigs,” and beliefs that the vaccines were developed to get rid of BIPOC individuals [20, 21, 23–26]. This also included a lack of trust in pharmaceutical companies specifically [25], and this was also mentioned in the present study, particularly Johnson & Johnson. While ideas were shared previously regarding what the vaccine would do to one’s body (e.g., nano bytes inserted into the body, DNA modification) [20, 25] our focus group discussions highlighted additional beliefs centered on the vaccines magnetizing individuals’ blood, and how its construction alters one’s DNA. This provides additional details about people’s concerns about the vaccines.

The importance of God, and acknowledging a spiritual component related to health is key in reaching the rural African American population in ENC. This is not surprising given that churches are important resources in rural communities for socialization, emotional support, and more, with pastors being trusted individuals. Prior research has examined belief in God or religiosity as associated with mistrust and/or intent not to vaccinate [27, 28]. The present study contributes to the literature by providing a different perspective on those who consider themselves religious and believe in God and their views regarding vaccination, beyond a binary association. As expressed by some participants who believed in God, this did not deter them from getting vaccinated, given that God provided man with common sense. Thus, people had a choice – to vaccinate or not vaccinate and everyone’s choice had to be respected. This also emphasized Dong et al.’s (2022) finding that vaccination was a choice that needed to be respected [20].

The present study had limitations that should be considered in future research. The sample included predominantly African American ENC residents, however, we only had one participant who identified as Hispanic or Latino. Given the current study setting, recruiting Hispanic/Latino individuals is important given the growth of this population in rural ENC. It is possible that cultural differences may have arisen and provided different perspectives on vaccination and messaging that could have contributed to the literature further such as work done by Perez et al. (2022) [29]. Second, one of the focus groups included fifteen participants. Six to twelve or a maximum of ten participants is generally the recommended size of focus groups [30, 31]. It is possible that given the large size of one focus group, we did not collect the responses of some participants who were perhaps more cautious about engaging in conversation with a large group. The authors’ field notes confirmed that the larger group was challenging for some people to participate in and despite their efforts to engage others, some individuals did not want to speak.

## Conclusion

Too often vulnerable communities are told what they should know, versus being asked what they think about certain health-related topics and how they think the information would be best received. Given the prolonged engagement of the authors in the community and rapport built with stakeholders and gatekeepers, the participants were comfortable in sharing their thoughts regarding the COVID-19 vaccines. The present study contributes to the knowledge regarding vaccine hesitancy and messaging by providing new insights that can inform the best ways to promote vaccine uptake among rural diverse adults. In sum, results from this study focus on at-risk ENC residents who have concerns about the COVID-19 vaccines. Such concerns along with reasons provided to seek vaccination could inform how health professionals can best address patient concerns and better understand their perspectives regarding vaccine uptake. Findings can also help inform campaigns that can work to effectively communicate with diverse and rural populations about COVID-19 vaccines. Although the present study focused on COVID-19, this vaccine research can be applied and has implications for other vaccine research, especially those considered more “recently” developed vaccines with uptake challenges among rural populations in the southeast [32, 33].

## Data Availability

The data are not publicly available due to sensitivity of human subject data and are available from the corresponding author upon reasonable request.
